# Modeling defects and plasticity in MgSiO_3_ post-perovskite: Part 2—screw and edge [100] dislocations

**DOI:** 10.1007/s00269-015-0763-8

**Published:** 2015-07-19

**Authors:** Alexandra M. Goryaeva, Philippe Carrez, Patrick Cordier

**Affiliations:** Unité Matériaux et Transformations, UMR CNRS 8207, Université de Lille1, Bat C6, 59655 Villeneuve d’Ascq Cedex, France

**Keywords:** MgSiO_3_ post-perovskite, D″ layer, Screw dislocations, Edge dislocations

## Abstract

In this study, we propose a full atomistic study of [100] dislocations in MgSiO_3_ post-perovskite based on the pairwise potential parameterized by Oganov et al. (Phys Earth Planet Inter 122:277–288, 2000) for MgSiO_3_ perovskite. We model screw dislocations to identify planes where they glide easier. We show that despite a small tendency to core spreading in {011}, [100] screw dislocations glide very easily (Peierls stress of 1 GPa) in (010) where only Mg–O bonds are to be sheared. Crossing the Si-layers results in a higher lattice friction as shown by the Peierls stress of [100](001): 17.5 GPa. Glide of [100] screw dislocations in {011} appears also to be highly unfavorable. Whatever the planes, (010), (001) or {011}, edge dislocations are characterized by a wider core (of the order of 2**b**). Contrary to screw character, they bear negligible lattice friction (0.1 GPa) for each slip system. The layered structure of post-perovskite results in a drastic reduction in lattice friction opposed to the easiest slip systems compared to perovskite.

## Introduction

The lowermost 200–300 km of the Earth’s mantle located above the core–mantle boundary (CMB), also known as D″ layer, is a very complex region (Wookey and Dobson 2008) characterized by strong lateral heterogeneity, pronounced seismic anisotropy and several regions of much reduced seismic velocities (ultra-low-velocity zones, ULVZs). Being at the interface between the core (made of a liquid iron alloy) and the silicate minerals of the mantle, the CMB represents one of the most important boundary layers of the Earth (with for instance a density contrast which exceeds the one between the crust and the atmosphere). As such, its physical properties are critical in determining the heat exchange between the core and the mantle and for the dynamics of mantle convection. The discovery of a transition of MgSiO_3_ perovskite (bridgmanite) to the post-perovskite phase (Murakami et al. 2004; Oganov and Ono 2004) at conditions close to those expected for the D″ region has opened new perspectives to understand the geophysics and geochemistry of the deep mantle. Among all physical properties, the thermal and rheological properties of the post-perovskite appear to be most influential. Indeed, the surface of the core is assumed to be essentially isothermal (Braginsky and Roberts 1995). Variations in temperature and heat flow in the CMB are thus mainly the consequence of mantle circulation patterns, strongly influenced by the rheological properties. Assuming the presence of a low-viscosity phase in the CMB has several major dynamical implications as discussed by Nakagawa and Tackley (2011) including a substantial increase in the heat flux from the core and of the overall convective vigor.

A few recent experimental and theoretical studies have raised the possibility that the post-perovskite would contribute to lowering the viscosity of the lowermost mantle. Deformation experiments performed on analog material CaIrO_3_ have shown evidence for viscosity reduction associated with the transformation from perovskite to post-perovskite (Hunt et al. 2009). Grain size reduction during phase transformation could be at the origin of this effect, possibly rendered more important by a sluggish grain growth in the post-perovskite phase (Yoshino and Yamazaki 2007). Moreover, first-principles calculations of atomic diffusion (Ammann et al. 2010) have shown that the mobility of cation vacancies in the post-perovskite phase is highly anisotropic (~8 orders of magnitude difference in diffusion coefficient). Assuming significant crystal-preferred orientation of post-perovskite, the existence of a fast diffusion direction would render it ~3 orders of magnitude weaker than that of perovskite. A key ingredient in Ammann and coworkers’ model is the development under dislocation creep of strong CPO resulting in an alignment of the [100] crystal axis in the shear direction.

In the present study, we propose to use full atomistic modeling to assess the ability of post-perovskite to deform by dislocation glide at 120 GPa. We follow the strategy proposed in Hirel et al. (2014). It consists in focusing on the easiest slip systems in the structure to place a lower bound on the rheological properties. Dislocations with screw character are first modeled. Since screw dislocations are not geometrically bound to glide in any specific plane, identifying the planes where they face the lowest lattice friction provides strong constraints on the easiest slip systems. In Hirel et al. (2014), this procedure is applied to [100] and [010] glide in MgSiO_3_ perovskite. It is shown that increasing pressure in the range 30–140 GPa induces a strong increase in lattice resistance to dislocation glide. In post-perovskite, the slip systems involving the shortest [100] Burgers vector and the [001](010) system within the glide plane cutting only Mg–O bonds are characterized with the lowest *γ*-surface energies (see Part 1, Goryaeva et al. 2015). Considering the potential importance of CPO involving alignment of [100] (and their potential role in explaining the D″ anisotropy, see Tsuchiya et al. 2004 and Wetzcovitch et al. 2006), we focus here on the modeling of dislocations with [100] Burgers vector.

## Computational details

A dislocation represents a linear defect with a translational symmetry characterized by a dislocation line **l**, and a Burgers vector **b** (the elementary amount of shear carried by the dislocation). These two vectors are parallel in case of screw dislocations and perpendicular in case of edge dislocations (Hirth and Lothe 1982). The long-range displacement field produced by dislocations can be described using continuum models, which, however, fail near the dislocation core where elastic theory breaks down. Atomic scale modeling is a powerful technic able to provide direct information about the dislocation core structure what is essential, for example, for understanding the anisotropy of plastic shear.

### Screw dislocations

#### Atomic systems and dislocation core energy

**Fig. 1:**
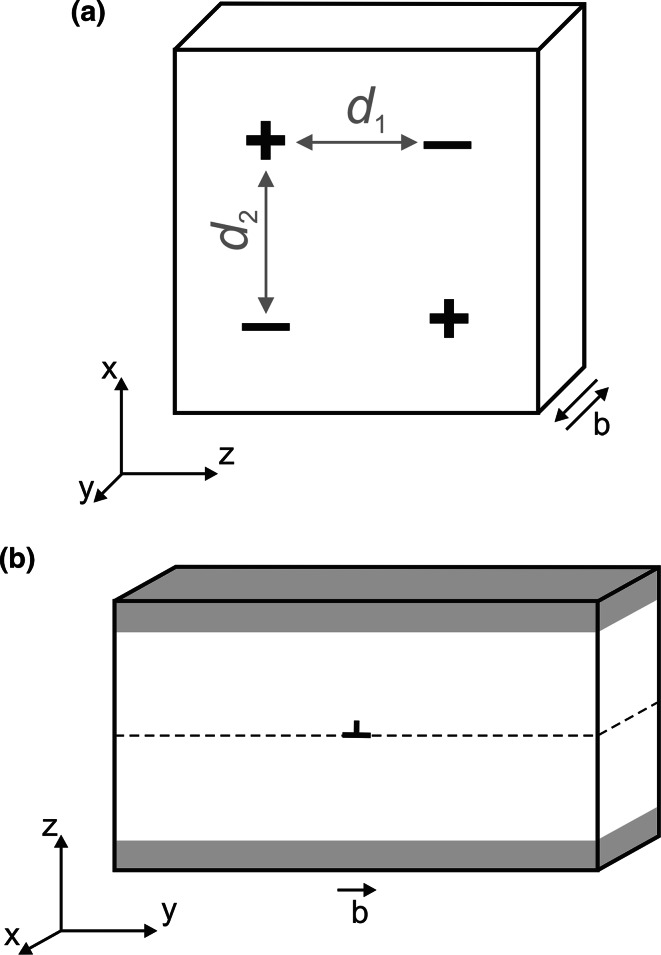
Geometry of atomic systems used for modeling of screw (**a**) and edge (**b**) dislocations. Simulation cells with quadrupole arrangement of screw dislocations (**a**) are fully periodic; screw dislocations with positive and negative Burgers vectors are shown with “+” and “−” signs, respectively. Edge dislocations are designed in atomic systems with cluster periodicity (**b**) such as atoms at the *top and bottom*, shown as *shaded areas*, are kept fixed; *dashed line* in the middle of the cell corresponds to the glide plane

In this work, screw dislocations are modeled within a quadrupole arrangement embedded in a fully periodic atomic array (Fig. 1a). Such a cell contains two dislocations with **b** = [100] and two dislocations with **b** = [${\overline {1}}00$]. This geometry allows to cancel the long-range displacement field produced by a single dislocation (Cai 2005). It ensures that interaction of dislocations remains only at a quadrupolar level and that the net force on each core is zero due to the periodic arrangement (Bigger et al. 1992). For such atomic configuration, the energy of a dislocation core *E*_c_ per Burgers vector **b** unit length can be extracted from the equation (Ismail-Beigi and Arias 2000):

1 $$E = E_{\text{c}} + E_{\text{el}} = E_{\text{c}} \left( {r_{\text{c}} } \right) + \frac{{\mu b^{3} }}{4\pi }\left[ {\ln \left( {\frac{{d_{1} }}{{r_{\text{c}} }}} \right) + A\left( {\frac{{d_{1} }}{{d_{2} }}} \right)} \right],$$

where *E* corresponds to the energy of a straight dislocation line (defined as the total energy of the atomic system once the energy of the perfect crystal is subtracted) which includes the elastic term *E*_el_ and the energy *E*_c_(*r*_c_) of a dislocation core with radius *r*_c_ (commonly taken equal to 2**b**) where elastic theory breaks down; *μ* is an anisotropic shear modulus depending on the elastic constants; *d*_1_ and *d*_2_ are equilibrium distances between the dislocations (Fig. 1a); $A\left( {\frac{{d_{1} }}{{d_{2} }}} \right)$ is a coefficient which includes all dislocation pairwise interactions and which depends only on the $\frac{{d_{1} }}{{d_{2} }}$ ratio. All simulation cells are designed in such a way that this ratio remains at the same value: 1.0071. Thus, all dislocations are equidistant, and the $A\left( {\frac{{d_{1} }}{{d_{2} }}} \right)$ value is constant for all supercells. Relying on these conditions, Eq. (1) is applied to evaluate the dislocation core energy using the energies *E* computed for ten different simulation cells, with sizes along *x* and *z* from ~145 to ~365 Å (8640–54,000 atoms). All atomic systems are as thin as a single **b** along *y* which ensures the dislocation lines to be straight and infinite by application of periodic boundary conditions. Simulation cells are oriented in such a way that **b** = [100] is aligned with *y* (Fig. 1a) and crystallographic directions [010] and [001] are aligned either with *x* or *z* depending on the glide plane of interest. The choice of cell orientations is driven by convenience to use the same atomic configurations for dislocation glide modeling as described below.

Initial configurations of screw dislocations are designed by imposing four isotropic displacement fields in a perfect crystal (Hirth and Lothe 1982). Then, structural relaxation (using the computational setup described below) yields dislocation core configurations counting anisotropic effects. From the relaxed atomistic configurations, one can easily compute the relative displacement of atoms near the dislocation core, also called disregistry. It will further be shown that most of disregistries can be analyzed with the help of the following *S*(*x*) function (Peierls 1940):

2 $$S\left( x \right) = \frac{b}{2} + \frac{b}{\pi }\arctan \left( {\frac{x}{\zeta }} \right) ,$$

where **b** is the Burgers vector and *ζ* is a half-width of the dislocation core. The derivative d*S*(*x*)/d*x* corresponds to the density of Burgers vectors, *ρ*(*x*), in a given plane, and its full width at half maximum defines the width of the dislocation core, i.e., 2*ζ*.

#### Dislocation glide

Dislocation motion is triggered by applying a simple shear strain to the simulation cell in order to increase the shear stress in a glide plane. The force acting on the dislocation is then given by the Peach–Koehler equation (Peach and Koehler 1950):

3 $${\mathbf{F}}_{{\mathbf{l}}} = \left( {\sigma \cdot {\mathbf{b}}} \right) \times {\mathbf{l}},$$

where **F**_**l**_ is a force acting on a unit length of a dislocation line **l**, *σ* is the applied stress tensor resulting from straining the cell, and **b** is the Burgers vector. All atomic systems are oriented in such a way that the dislocation line **l** is along *y* and to promote glide in a plane normal to the cartesian direction *x*. This means that the dislocation will move along *z* (the corresponding crystallographic directions depend on the glide plane of interest). In order to initiate dislocation glide, *ε*_*xy*_ is gradually increased. To ensure quasi-static loading, after applying a shear strain increment (of the order of 1 × 10^−4^), the deformed atomic configuration is optimized according to the procedure described in the computational setup. The Peierls stress (the critical stress required to move a straight dislocation at 0 K) is evaluated as the derivative of the potential energy of the system with respect to the applied shear displacements. Its values estimated for different glide planes describe anisotropic lattice friction in a material.

### Edge dislocations

A dipole arrangement of edge dislocations with positive and negative Burgers vectors in a fully periodic supercell is commonly used for metals (Chang et al. 1999; Bulatov et al. 1999; Chang et al. 2002). However, this configuration is not always appropriate, especially for materials with complex crystal chemistry. In the post-perovskite structure, edge dislocations lying on the same glide plane with positive and negative Burgers vectors^1^ have different geometries. Thus, in our case, a fully periodic dipole cell containing two geometrically identical glide planes would incorporate two different dislocation cores. Reciprocally, a periodic dipole configuration containing two identical dislocation cores of opposite sign would not be charge neutral. This prevents us from using a fully periodic system for edge dislocation modeling. Therefore, we chose to model single-edge dislocation in a cluster-like configuration (Osetsky and Bacon 2003; Monnet and Terentyev 2009; Hirel et al 2014). The supercells are designed to be fully periodic along the directions of the dislocation line (*x*) and of the Burgers vector (*y*). Along the *z* direction, the cluster approach is applied (Fig. 1b), i.e., the atoms at the bottom and at the top of the supercell (shown as shaded area in Fig. 1b) are fixed to their regular positions, and atomic relaxation is allowed for the rest of the crystal. The width of the layer where atoms are fixed (along *z*) is equal to the potential short-range cutoff distance, i.e., to 12 Å in our case. This allows to mimic an infinitive perfect crystal and to avoid ineligible interactions between periodic replicas. For such a system, an edge dislocation is constructed by building and joining two supercells, one of them containing an atomic extra half-plane (the top one for dislocation with positive **b** and the bottom one for negative **b**). Structural relaxation of the joint atomic array gives rise to the edge dislocation (Osetsky and Bacon 2003; Bulatov and Cai 2006) with a Burgers vector aligned with *y* and a dislocation line along *x*. All atomic systems are constructed in such a way that charge neutrality and stoichiometry are preserved. In this geometry, the core structure of a dislocation and the Peierls stress can be affected by the interaction of the dislocation with its periodic images if dislocations are too close to each other, i.e., if the supercell is not large enough. Thus, the size of the supercell is gradually increased until convergence is reached. Typical simulation supercell sizes are ~250 Å along *y*, ~140 Å along *z*, and as a single unit cell parameter along *x*. Such cells contain the order of 30,000–50,000 atoms depending on the unit length along *x*.

Based on the same principles as screw dislocation glide, conservative motion of edge dislocations along *y* is caused by shearing the simulation cell to apply *ε*_*yz*_ with 1.5 × 10^−5^ increments.

### Computational setup

In this study, atomistic modeling of dislocations is performed in MgSiO_3_ post-perovskite using the Buckingham form of a pairwise potential with the parameterization derived by Oganov et al. (2000) for MgSiO_3_ perovskite. In the first part of this study, we have validated the use of this potential for modeling *γ*-surfaces of MgSiO_3_ post-perovskite (Goryaeva et al. 2015). Molecular statics simulations are carried out at *P* = 120 GPa and constant volume using the program package LAMMPS (Plimpton 1995), which relies on Ewald summation methods for Coulombic interactions. Optimization is performed using a conjugate-gradient algorithm followed by a Hessian-free truncated Newton algorithm until the maximum force on an atom drops below 10^−9^ eV/Å (1.602 × 10^−18^ N).

## Results

### Screw dislocations

**Fig. 2:**
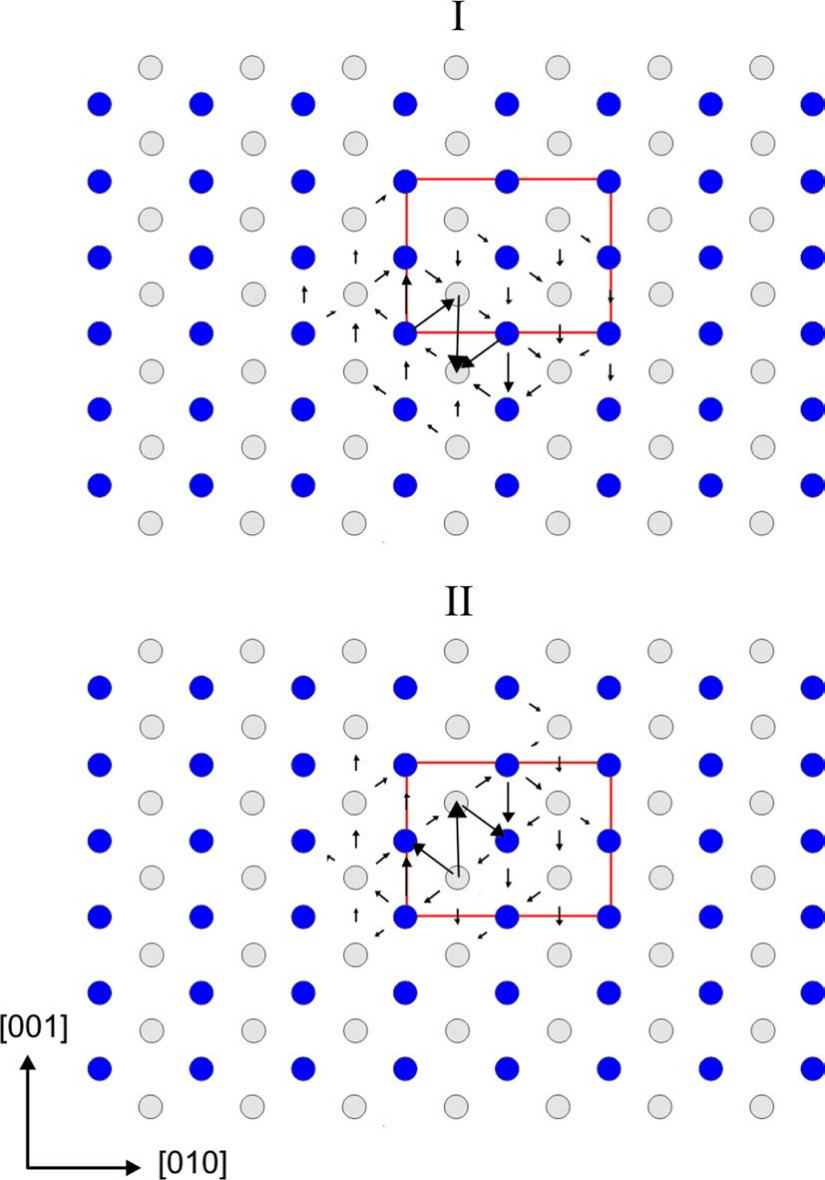
Core structures of the [100] screw dislocations viewed along the burgers vector direction: **I** with the dislocation line located at (∞, ¼, 0); **II**—at (∞, ¼, ½). The anion sublattice is left out. Si atoms are shown with *dark blue balls*, Mg atoms—with *gray balls*; the unit cell—with *red rectangle*. The *arrows* between atoms correspond to the [100] component of the relative displacement of the neighboring atoms produced by the dislocation. The length of the *arrows* is proportional to the magnitude of these components

**Fig. 3:**
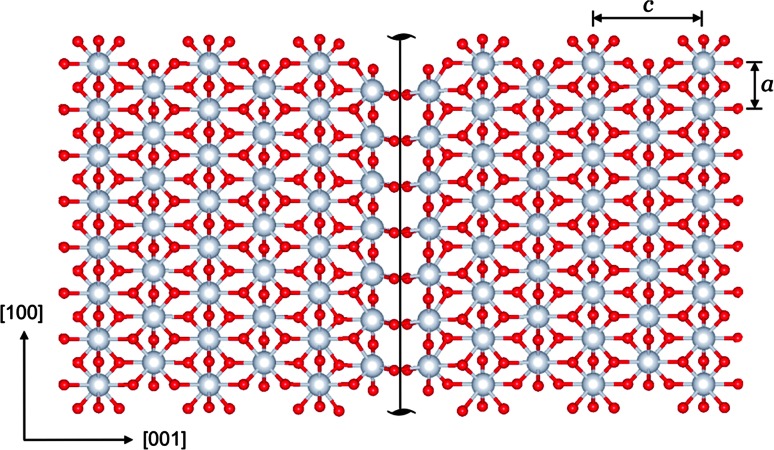
Local structure produced by a screw dislocation in MgO layer

**Fig. 4:**
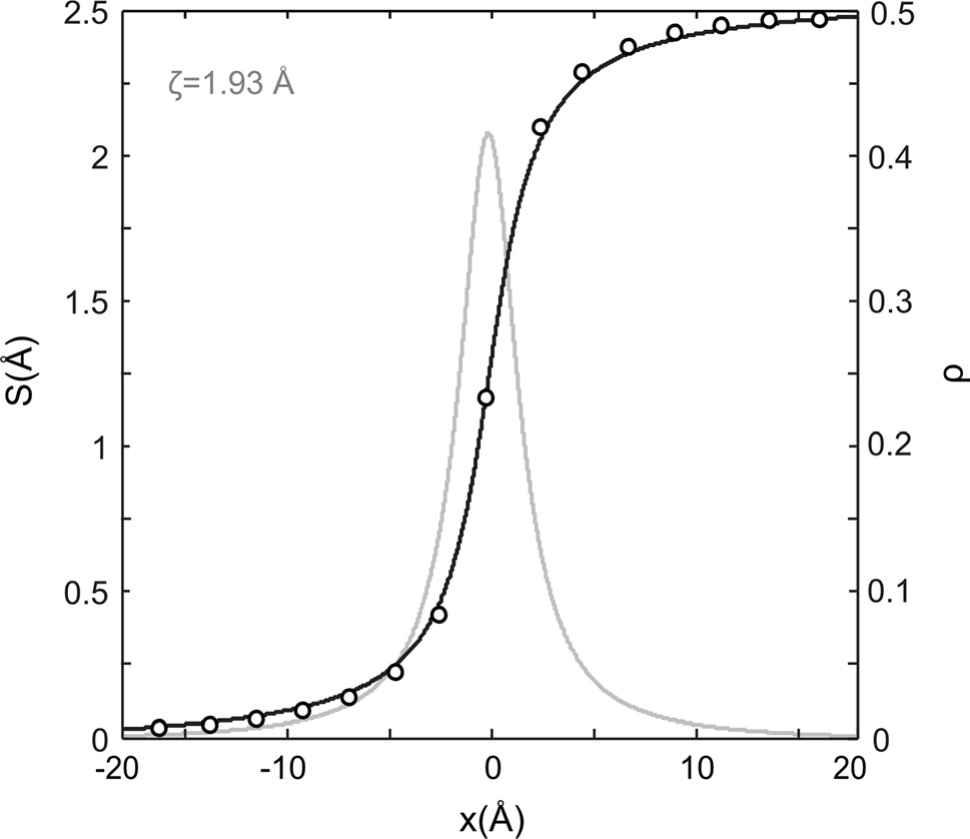
Disregistry function *S*(*x*) and the [100] Burgers vector density *ρ*(*x*) of the stable screw dislocations (**I**) and (**II**) computed for the cation sublattice. Evaluated value of the dislocation core half-width *ζ* is given in the plot

The shortest Burgers vector [100] is found to produce two possible geometries of stable screw dislocation cores. Figure 2 shows a visualization of these dislocations based on differential displacement (DD) maps. For screw dislocations, all displacements are parallel to the line and hence cannot be visualized in views parallel to this direction which are adapted to show the core. In DD maps, the relative displacement between neighboring atoms, which is produced by the dislocations and which is perpendicular to the plane of view, is represented by an arrow between those two atoms. The lengths of the arrows scale to the amplitude of the displacement vector. Both dislocations (I) and (II) exhibit pure screw cores without any edge component. The dislocation line (I) located at (∞, ±¼, 0) is characterized by a compact planar core with a little spreading in (011). The dislocation line (II) located at (∞, ±¼, ½) is very similar, but it spreads in ($0{\overline {1}}1$). In fact, these dislocations represent a mirror reflection of each other. Locations of the dislocation lines coincide with the positions of [100] screw axis 2_1_ between two Mg atoms. Superposition of these elements creates a local structure along the dislocation line similar to the action of a 2 axis instead of the original 2_1_ (Fig. 3). The dislocation cores tend to spread toward the empty trigonal channels located between Si- and Mg-layers. The spreading of the cores is limited by two Si-layers, and the distortion produced by the dislocation core notably affects interconnection of Mg-polyhedra only. Figure 4 shows the atomic disregistry *S*(*x*) along <011> corresponding to this [100] screw dislocation calculated for the cations sublattice. The Burgers vector density *ρ*(*x*) (derivative of the disregistry) is also presented. These functions are identical for dislocations (I) and (II). The evaluated half-width of the dislocation core *ζ* is 1.93 Å (Fig. 4).

**Fig. 5:**
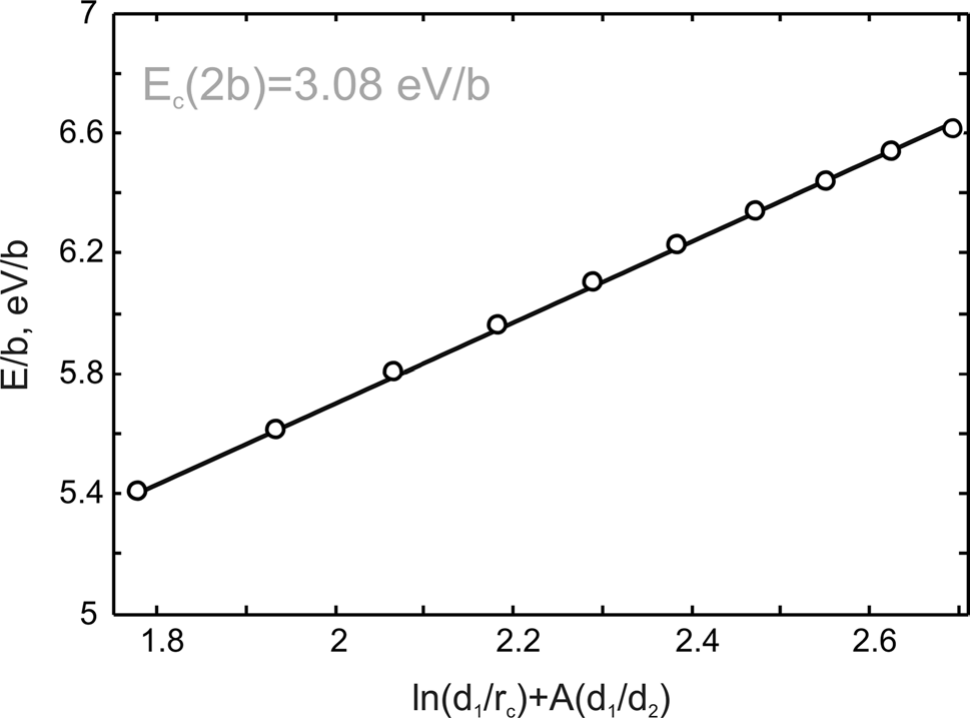
Evolution of the [100] screw dislocations total energy (including elastic energy and the dislocation core energy) as a function of ln(*d*_1_/*r*_c_) + *A*(*d*_1_/*d*_2_)

The dislocation core energy is also identical for the screw dislocations (I) and (II), ultimately confirming their equivalence. The minimized energy of atomic systems with different sizes is shown in Fig. 5 as a function of ln(*d*_1_/*r*_c_)+*A*(*d*_1_/*d*_2_) [see Eq. (1)]. The energies follow a straight line as predicted by the elastic theory. The calculated value of the *A* coefficient including all the effects of the infinite Ewald-like sums of dislocation interactions is −0.88548. The linear fit provides the dislocation core energy *E*_c_(2*b*) = 3.08 eV/*b* and the anisotropic shear modulus *μ* = 167 GPa. The value *μ* estimated from the elastic constants is 173 GPa which compares well with the modulus obtained from the linear fit.

**Fig. 6:**
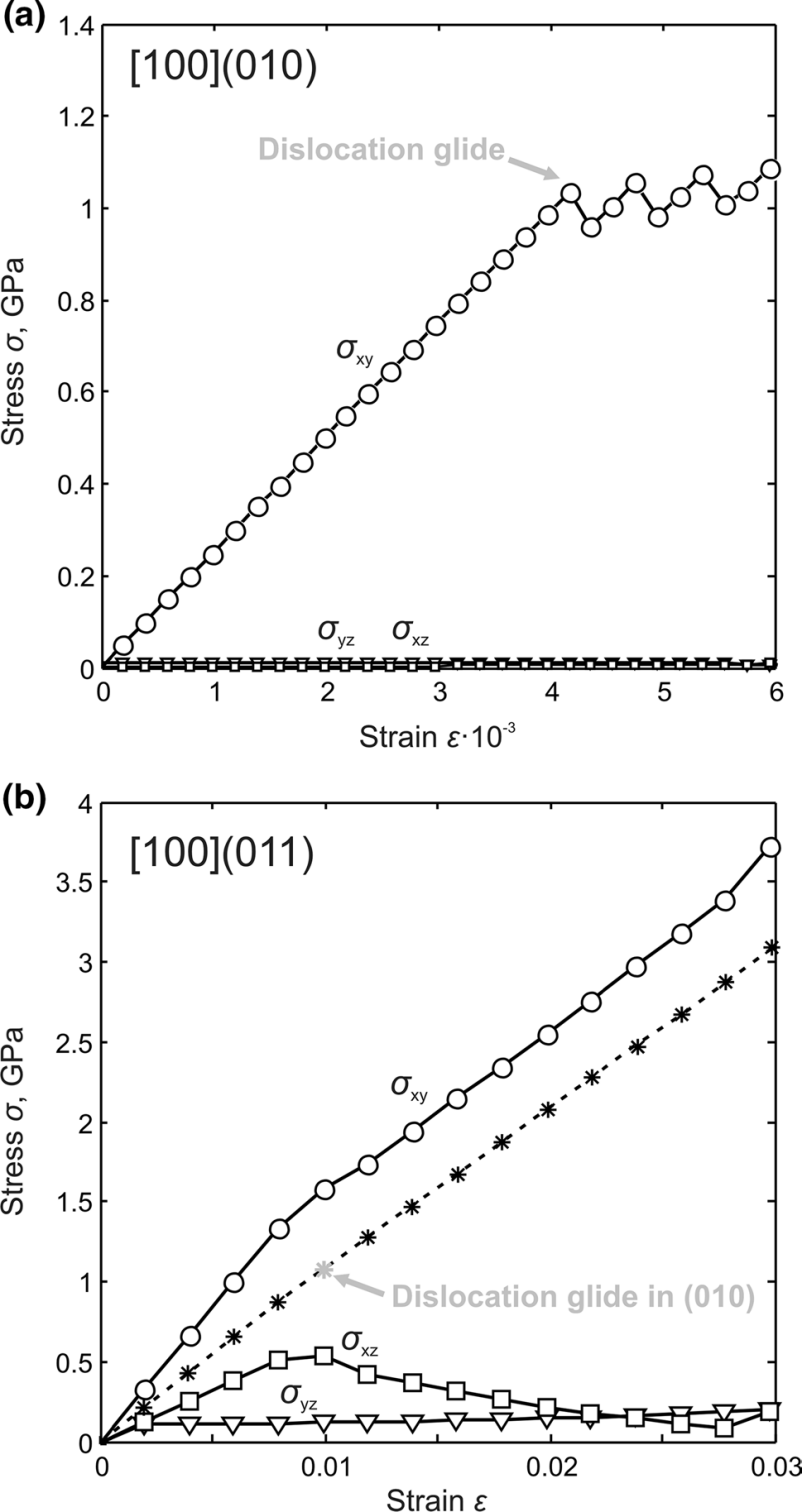
Evolution of the *σ*_*xy*_, *σ*_*xz*_ and *σ*_*yz*_ stress components **a** while applying a simple shear to an orthorhombic cell based on vectors [010], [100] and [001] [in order to trigger [100] screw dislocation glide in (010)] and **b** a monoclinic supercell built on vectors [059], [100] and [$01{\overline {1}}$] in order to activate [100](011) system. For the monoclinic configuration (**b**), the resolved stress on (010) is shown with the *dashed line*

**Fig. 7:**
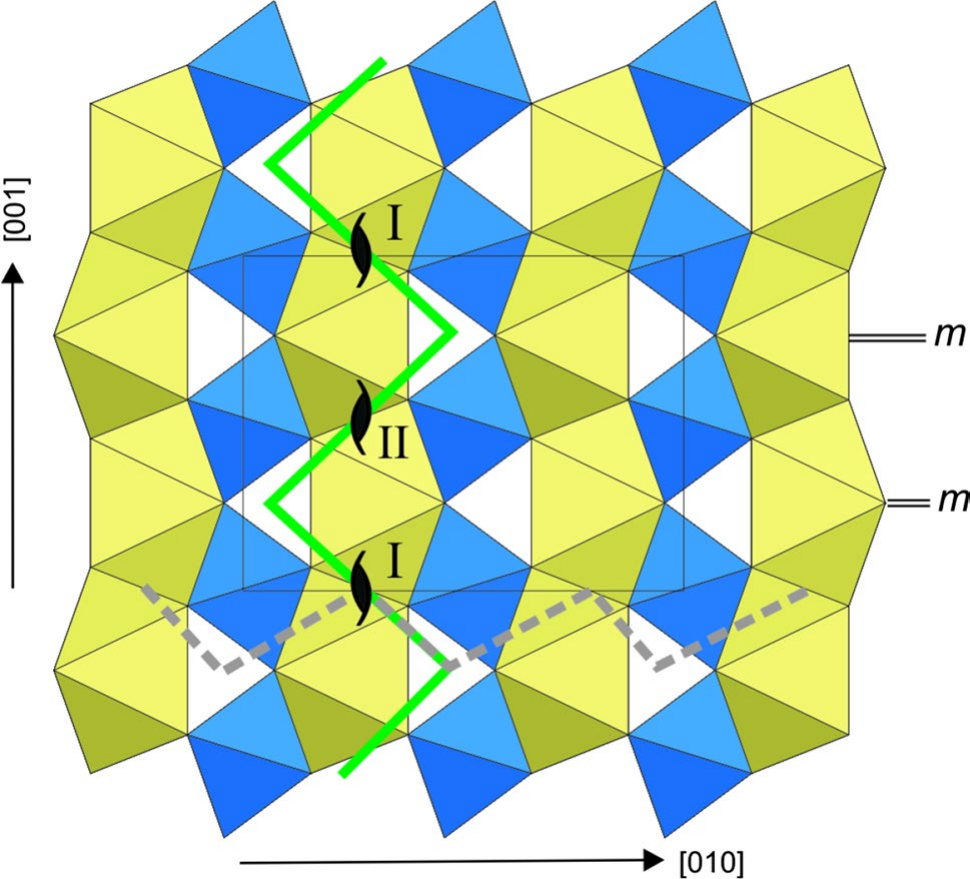
Observed paths of [100] screw dislocations glide in (010) and (001). The easiest (010) glide is shown with a *solid green line*; the difficult (001) glide—with a *dashed gray line*. Location of stable screw dislocations (**I**) and (**II**) in the structure are given with “screw” signs; location of mirror planes *m*_001_ between these dislocations are indicated as “*m*”

Glide of the [100] screw dislocations is triggered by applying a simple shear in order to increase stress in a glide plane of interest. When stress reaches a critical value, i.e., the Peierls stress value, the dislocations start to glide. Monitoring the system stress field and the disregistry function of the dislocation allows to determine the Peierls stress value (Fig. 6a). Motion of all dislocations within the quadrupole system starts simultaneously. Dislocations with opposite Burgers vectors move toward each other and eventually annihilate. Lattice friction (described by the Peierls stress) displays a highly anisotropic behavior: Peierls stresses are 1 and 17.5 GPa for glide in (010) and (001), respectively. Figure 7 shows that glide in (010) and (001) occurs through successive jumps forming zigzag paths. The remarkably easy [100](010) glide occurs strictly *within* a Mg–O layer.

Modeling dislocation glide in (011), i.e., in the plane where the dislocation core exhibits a slight tendency for spreading, requires rebuilding a simulation cell in such a way that lattice vectors **a**_**1**_, **a**_**2**_ and **a**_**3**_ are aligned with [059], [100] and [$01{\overline {1}}$], respectively. There is a monoclinic angle 89.95° between [059] and [$01{\overline {1}}$] which is very close to an orthogonal configuration. Applying a strain component *ε*_xy_ to the monoclinic cell (characterized with elastic tensor containing 13 independent components) results here in an increase in several stress components (Fig. 6b). During the deformation, the *σ*_*xy*_ component, needed to activate glide in (011), increases much faster than the residual *σ*_*xz*_ and *σ*_*yz*_ components (Fig. 6b). However, whereas the system geometry is built to maximize the resolved shear stress in (011), the close orientation of (010) results in a nonzero resolved shear stress in this plane as *σ*_*xy*_ increases. Due to the low lattice friction of the [100](010) system, it turns out that the dislocations always start gliding in (010) instead of in the desired (011) plane when the resolved stress on (010) (resulting from *σ*_*xy*_ and *σ*_*xz*_) reaches the critical value of 1 GPa (Fig. 6b). A lowest bound of the Peierls stress needed for glide in (011) can be evaluated from the resolved stress on (011) based on calculations for [100](001) glide. For this orientation, the resolved shear stress on (010) is null, preventing dislocations to glide in this plane. The resolved stress on (011) reached 10.4 GPa without promoting glide in (011). Our simulations clearly demonstrate that for [100] screw dislocations, the Peierls stress in (010) is much smaller than in (011) (*σ*_p_ > 10.4 GPa) and in (001) (*σ*_p_ = 17.5 GPa).

### Edge dislocations

In this study, we mainly focus on [100](010), [100](001) and [100](011) edge dislocations within glide planes characterized with the lowest *γ*-surface energies, i.e., corresponding to *z*_010_ = 0.7, *z*_001_ = 0.65 and *z*_011_ = 0.42, respectively^2^ (see Part 1, Goryaeva et al. 2015).

**Fig. 8:**
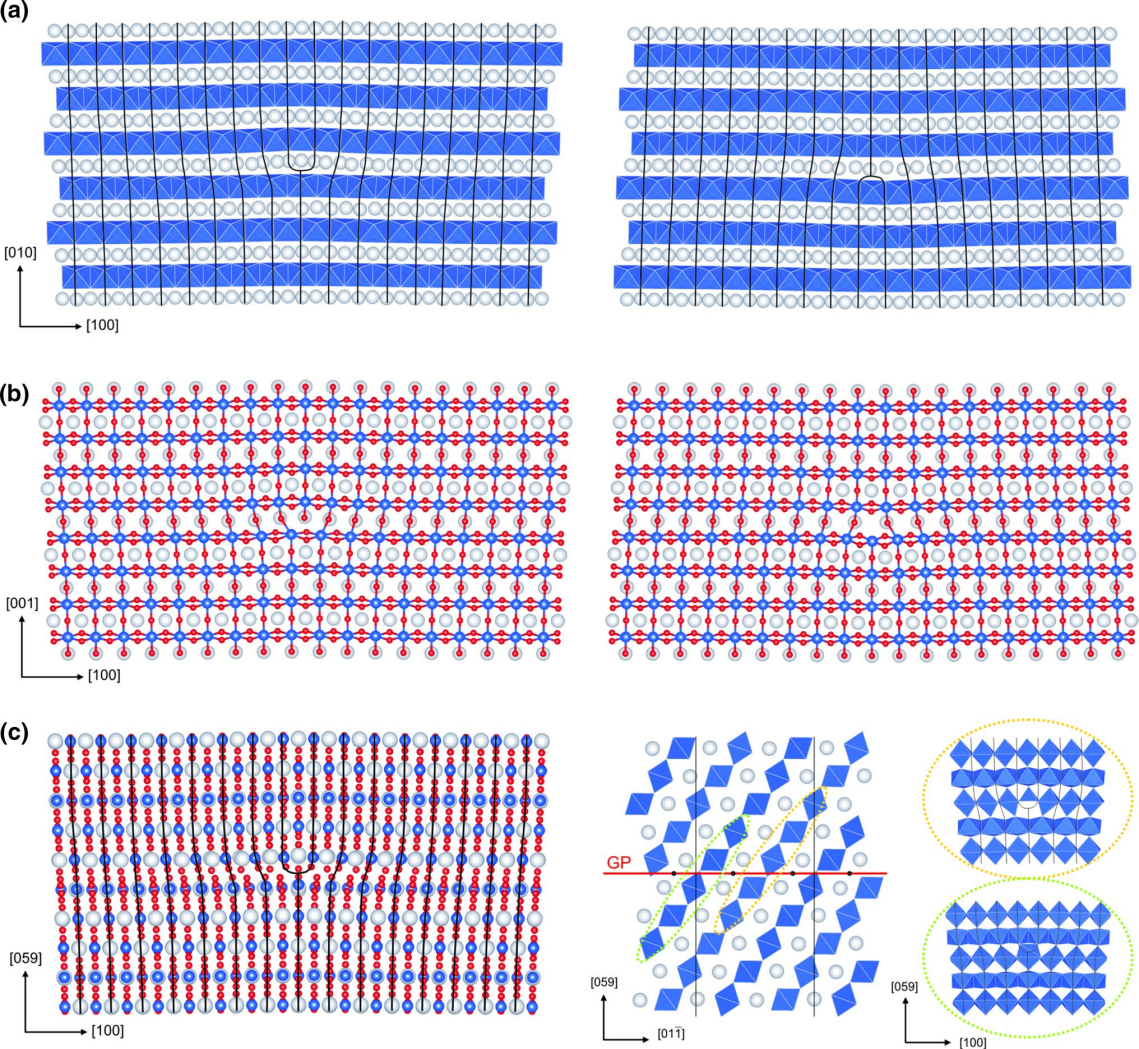
Atomic structure of the relaxed [100](010) (**a**), [100](001) (**b**) and [100](011) (**c**) edge dislocation cores. *Right part* of (**c**) shows location of (011) glide plane in the structure with respect to Si-octahedra

Due to the location of the (010) glide plane at *z*_010_ = 0.7 (affecting only Mg-polyhedra), all Si atoms in the dislocation core keep octahedral coordination (Fig. 8a). The length of [100] Burgers vector **b** = 2.521 Å is equal to the size of a single SiO_6_ octahedron. Thus, in the [100](010) dislocation cores, there is a local disturbance of the regular chessboard pattern of Si-octahedra produced by the *C*-lattice. Edge dislocation with positive Burgers vector is characterized with a lack of O for Mg coordination in the dislocation core, which results in the presence of sixfold and sevenfold coordinated Mg along the dislocation line. On the contrary, in a dislocation core with negative Burgers vector, there are extra O atoms increasing coordination of some Mg atoms located along the dislocation line up to 10. Both dislocation cores are very symmetric.

Edge dislocations lying on (001) plane (with *z*_001_ = 0.65) are also very symmetric. The chosen (001) glide plane is located in the structure in such a way that all Mg-polyhedra below this plane remain eightfold coordinated for both dislocations with positive and negative Burgers vectors, whereas coordination of Mg above the glide plane is going to be incomplete. Similar to [100](010), the edge [100](001) dislocations with positive Burgers vector retain the favorable octahedral coordination of Si (Fig. 8b). Mg atoms located in the dislocation core above the glide plane are sixfold and sevenfold coordinated. Inside the dislocation core with negative Burgers vector, there are Si atoms with incomplete fivefold coordination which also can be described as (5 + 2) while taking into account next two closest (~2.4 Å) O atoms (Fig. 8b). Above the glide plane, Mg atoms located along the dislocation line are sixfold coordinated. Thus, for the dislocation cores with positive and negative **b**, distortions of Mg-polyhedra are quite similar, while Si changes coordination only in the dislocation core with negative Burgers vector.

The chosen (011) glide plane located at *z*_011_ = 0.42 is the only plane considered in this work which has a symmetric position in the post-perovskite structure. This plane contains inversion centers (Fig. 8c), which results in identical core structures for dislocations with negative and positive Burgers vectors. Optimization of the dislocation core structure is performed employing supercells based on the same monoclinic vectors [100], [$01\overline {1}$] and [059] as for the [100](011) screw dislocation modeling. Inside these identical dislocation cores, there are two Si and four Mg atoms (per unit length [$01\overline {1}$]) which change their coordination. Two of these four Mg atoms are overbonded and have coordination 9, while other two Mg have only seven ligands. The distorted Si-polyhedra are located on different sides from the glide plane: One is above and another one is below (Fig. 8c). Incorporation of atomic extra half-plane above the glide plane leads to appearance of an extra O ligand for the Si atom below the plane and lack of one O ligand for the Si atom above the glide plane (Fig. 8c). The incomplete fivefold coordination of the latest is similar to the one observed in the [100](001) dislocation core with negative Burgers vector.

**Fig. 9:**
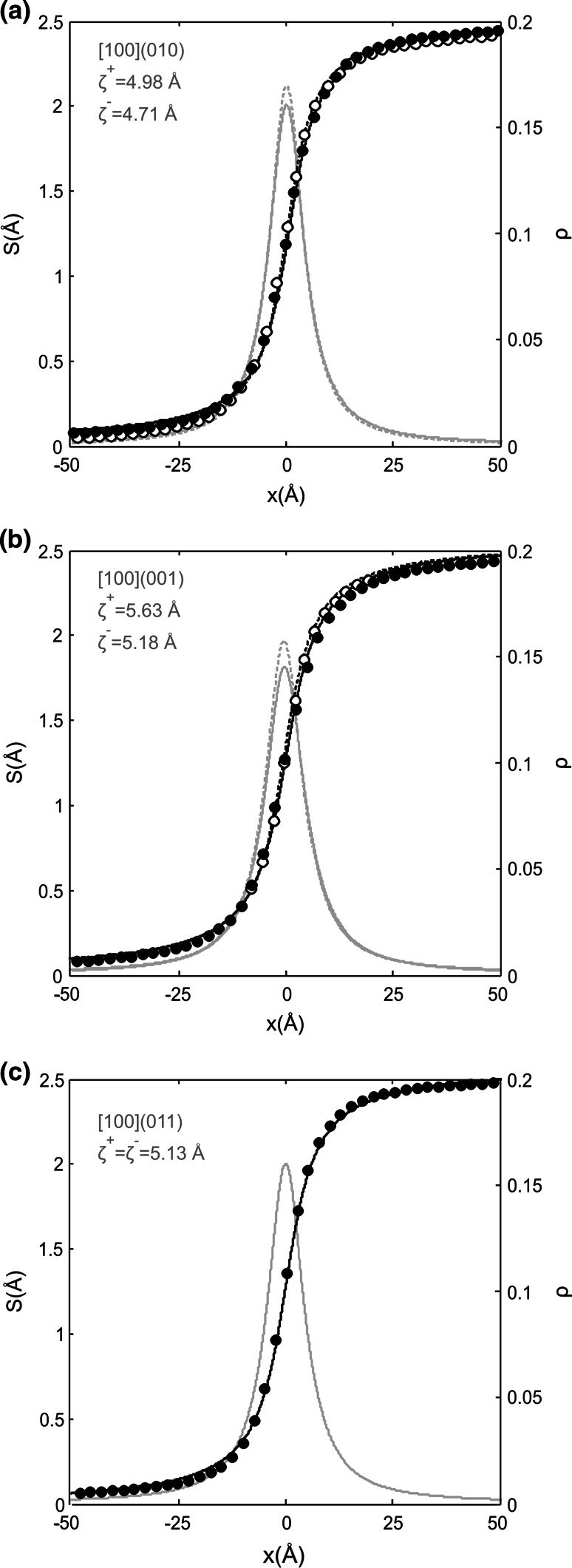
Disregistry functions *S*(*x*) and the Burgers vector density *ρ*(*x*) of the [100](010) (**a**), [100](001) (**b**) and [100](011) (**c**) edge dislocations computed for the cation sublattice. *Solid lines* correspond to dislocations with positive Burgers vector; *dashed lines*—with negative. In case of [100](011) (**c**), they coincide. Evaluated value of the dislocation core half-widths *ζ* is given in the plot

The computed disregistry functions *S*(*x*) and their derivatives *ρ*(*x*) have a symmetric shape for all edge dislocations described above, which was expectable from the observed dislocation core structures. All edge dislocations are characterized by the dislocation half-width *ζ* very close to 2**b** (Fig. 9a–c; Table 1) which is almost three times larger than for the [100] screw dislocations.

**Table 1:** Peierls stresses *σ*_p_ and dislocation core half-widths *ζ* calculated for all possible atomic configurations of [100] edge dislocations in post-perovskite. Different values of *z*_*hkl*_ correspond to different locations of glide planes in the structure described in detail in Part 1 (Goryaeva et al. 2015)

System		Positive **b** (above GP)		Negative **b** (below GP)	
		*ζ*, Å	*σ*_p_, GPa	*ζ*, Å	*σ*_p_, GPa
[100](010)	*z*_010_ = 0.7	4.98	<0.1	4.71	<0.1
	*z*_010_ = 0.4	4.13	7.6	4.13	1.0
	*z*_010_ = 0.55	4.46	2.6	3.42	17
[100](001)	*z*_001_ = 0.65	5.63	<0.1	5.18	~0.15
	*z*_001_ = 0.47	5.24	8	4.56	0.5
[100](011)	*z*_011_ = 0.42	5.13	~0.12	5.13	~0.12
	*z*_011_ = 0.49	6.00	5	4.43	5.5
	*z*_011_ = 0.54	4.29	6.5	5.49	12

The motion of the [100] edge dislocations is studied by applying a simple shear strain *ε*_*yz*_ in order to force a dislocation line lying along *x* to glide in the plane normal to *z*. As expected, edge dislocations are much more mobile than screw dislocations. Glide of [100](010) dislocations with both positive and negative Burgers vectors occurs at very low stresses *σ*_p_ < 0.1 GPa. The lattice friction of other dislocations is somewhat higher but still very low. Thus, the Peierls stresses estimated for the [100](001) edge dislocations with positive and negative Burgers vectors are 0.1 and 0.15 GPa, respectively. Clearly, this difference is mainly caused by different level of the Si-octahedra distortions in the dislocation cores. The [100](011) edge dislocations start gliding at *σ*_p_ ~ 0.12 GPa.

## Discussion

### Screw dislocations

The [100] screw dislocations in MgSiO_3_ post-perovskite are characterized by a narrow planar core spread in {011}. The half-width of the dislocation core *ζ* = 1.93 Å estimated from our full atomistic simulations is almost twice bigger than the half-width *ζ* = 1.03 Å of the corresponding screw dislocation provided by the Peierls–Nabarro (PN) model (Carrez et al. 2007). The apparent discrepancy between PN model and the atomistic calculations may come from the generalized stacking fault energy level used for the PN modeling (see Part I). Regarding the core energy, the full width of the dislocation core *r*_c_ = 3.86 Å is still less than *r*_c_ = 2**b**, employed for evaluation of the dislocation core energy. Relying on the real value *r*_c_ obtained from atomistic modeling, the dislocation core energy is *E*_c_(*r*_c_) = 2.72 eV/*b* (1.08 eV/Å) which is slightly lower than the value *E*_c_(2**b**) = 3.08 eV/*b* (1.23 eV/Å). The dislocation core energy can also be evaluated as $\eta \frac{{\mu b^{3} }}{4\pi }$ where *η* is a screening factor (Joos and Zhou 2001). Thus, energetic characteristics of a dislocation core are strongly dependent on elastic modulus *μ* and on the screening factor *η* which significantly varies for different materials. For instance, in metals this factor is commonly taken as 0.5 (Joos and Zhou 2001), while its value estimated for the post-perovskite is about 2.2. Thus, silicate post-perovskite is clearly characterized by a dislocation core energy which is larger than for metals. However, for ½[110] screw dislocations in MgO at 100 GPa, both elastic modulus *μ* = 195 GPa and screening factor *η* = 2 are close to those in MgSiO_3_ post-perovskite, and this results in comparable values for the dislocation core energy *E*_c_(*b*) = 1.28 eV/Å (Carrez et al 2015).

Significant differences in crystal chemistry between Mg–O and Si–O layers determine the dislocation core spreading limited by two neighboring Si-layers and the observed highly anisotropic lattice friction. Thus, estimated Peierls stress for dislocation glide in (001) is almost 18 times larger than in (010) plane parallel to the structural layering. Commonly, the easiest dislocation glide occurs in a plane where the dislocation core spreads (Hirth and Lothe 1982). However, the [100] screw dislocations in MgSiO_3_ post-perovskite rather tend to glide in (010) than in {011}. Nevertheless, the observed zigzag path of the [100](010) gliding (Fig. 7) explicitly reproduces the geometry of dislocation core spreading while switching from dislocation (I) spread in (011) to its symmetric replica (II) spread in ($01\overline {1}$) (Fig. 2). This phenomenon cannot be described as a cross-slip mechanism because the general direction of glide remains the same and clearly corresponds to (010). This remarkably easy glide occurs strictly *within* Mg–O layer which is correlated with the [100](010) *γ*-line at z_010_ = 0.7 cutting only Mg–O bonds. The observed motion in (001) plane matches the [100](001) *γ*-line at z_001_ = 0.65. Thus, in both cases, glide of [100] screw dislocations corresponds to the low-energy *γ*-lines.

The conservative screw dislocation motion was also examined for simulation supercells with cluster periodic boundary conditions similar to those designed for edge dislocation modeling (see “Computational details” section). These simulations provide the same dislocation core geometry and Peierls stresses of 1.1 and 17.5 GPa for [100](010) and [100](001), respectively. Modeling screw dislocation glide in (011) plane produces the same effect of activating [100](010) glide. These results compare well with the values computed using the fully periodic supercells, which shows this cluster cell geometry to be appropriate for dislocation modeling.

### Edge dislocations

The [100] edge dislocations lying on (010), (001) and (011) are consistently characterized by dislocation core half-widths *ζ* very close to 2**b** (Fig. 9a–c; Table 1) which is almost three times larger than for the compact [100] screw dislocations. Due to the peculiarities of the complex post-perovskite structure, the dislocation cores are different for configurations with positive and negative Burgers vectors, except for the [100](011) dislocations with symmetric location of the glide plane at z_011_ = 0.42 in the structure (Fig. 8c). The easiest dislocation glide is observed for the [100](010) edge dislocations at *σ*_p_ < 0.1 GPa. However, in contrast to screw dislocations, glide of edge dislocations in (001) and (011) occurs almost as easily as in (010).

Location of the glide planes in the structure was chosen based on the calculations of *γ*-surface energies (see Part 1, Goryaeva et al. 2015), i.e., glide planes of edge dislocations correspond to the planes with the lowest *γ*-energies. In order to confirm this choice ultimately, modeling of edge dislocations with other possible locations of glide planes in the structure has been performed. The simulations show that gliding in planes with the lowest *γ*-energies is always the easiest (Table 1). However, the stress values estimated for edge dislocations within glide planes of the highest *γ*-energies are not necessarily the largest. Moreover, the Peierls stress values vary considerably for dislocations of opposite sign lying on the same glide plane (Table 1). For instance, the (010) plane with z_010_ = 0.55 is characterized by the largest (010) *γ*-energy. However, for this plane, the Peierls stress of the edge dislocation with positive Burgers vector is smaller than the one of the dislocation based on the lower-energy plane (010) with z_010_ = 0.4 (Table 1). The same tendency is also observed for (001) and (011) planes. Generally, for these configurations, the Peierls stress is strongly influenced by crystal chemistry, and its largest values always correspond to the dislocations with the largest distortion of Si-polyhedra in the dislocation core. For the low-energy planes (with z_010_ = 0.7, z_001_ = 0.65 and z_011_ = 0.42), this effect is also observed, but it is not as significant as for the high-energy configurations.

### Implications

**Fig. 10:**
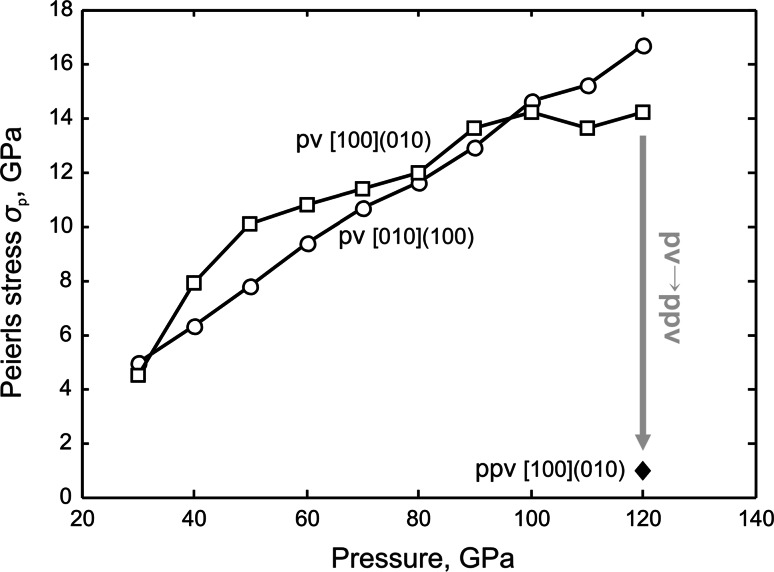
Dependence of the Peierls stress *σ*_p_ related to the easiest screw dislocation glide in MgSiO_3_ perovskite (pv) and post-perovskite (ppv) phases on pressure corresponding to lower mantle. Values for the pv phase are taken from Hirel et al. (2014)

The atomic structure of [100] dislocations in MgSiO_3_ post-perovskite and its anisotropic lattice friction represent an issue of primary importance for understanding anisotropic plasticity of this mineral phase stable at the CMB. We find that glide of [100] dislocations is very easy in the (010) plane within the Mg–O layer. These results are in agreement with the TEM observations of [100](010) dislocations in deformed CaIrO_3_ post-perovskite analog (Walte et al.^2007^; Miyajima and Walte 2009). To go further and to highlight the importance of this effect, it is worth comparing with MgSiO_3_ perovskite (bridgmanite). A previous atomic scale study of MgSiO_3_ perovskite (Hirel et al. 2014) has shown that in this structure, lattice friction increases significantly over the pressure range of the lower mantle. In Hirel et al. (2014), the strategy was similar to the one used here, focusing on the easiest slip systems ([100](010) and [010](100) in bridgmanite) and employing the same potential model. Figure 10 compares the Peierls stresses of the easiest screw dislocations in bridgmanite as a function of pressure and in the post-perovskite. It is shown that the change of crystal structure which results in a layering is responsible for a drop in the lattice friction (considering the easiest slip systems of course). From a completely different perspective, our calculations support the suggestion from Ammann et al. (2010) (based on a theoretical study of anisotropic diffusion in post-perovskite) that the transition to the post-perovskite may be responsible for a weaker layer in the D″ layer.

## Conclusions

Atomic scale modeling directly provides essential structural information about screw and edge dislocation cores in MgSiO_3_ post-perovskite under 120 GPa confining pressure. The [100] edge dislocations are characterized by dislocation cores with a half-width very close to 2**b**. This is almost three times larger than the half-width of the compact cores of screw dislocations which show a slight tendency for spreading in {011}. Lattice friction opposed to the glide of [100] dislocations in MgSiO_3_ post-perovskite is highly anisotropic. Despite its narrow core structure, remarkably low values of Peierls stress (1 GPa) are found for the glide of [100] screw dislocations in (010), while glide in (001) requires stress value which is almost 18 times larger than in (010). Dislocation glide in {011} could not be activated while applying a simple shear; however, the Peierls stress for this system appears to be bigger than 10.4 GPa. The observed path of screw dislocation gliding in (010) and (001) reproduces low-energy *γ*-lines. Edge dislocations modeled within glide planes with the lowest *γ*-energies are characterized by notably lower stresses than those constructed for high-energy configurations. Thus, *γ*-surface calculations clearly give an idea about the most probable path of dislocation gliding. As expected, mobility of edge dislocations is much higher than that of screw dislocations, and the latter will control plastic behavior of MgSiO_3_ post-perovskite.

Comparison of our results with previous study of MgSiO_3_ perovskite (Hirel et al. 2014), based on similar simulation approach, clearly shows that monotonous increase in Peierls stress of bridgmanite will be followed by a dramatic drop after the phase transition to the post-perovskite phase, which consequently suggests the D″ located at the CMB to be weaker than the overlying mantle.

## Footnotes

1 Edge dislocation with positive Burgers vector corresponds to the configuration where atomic extra half-plane is located above the glide plane, while dislocations with negative Burgers vector—below the glide plane.

2 Here and hereafter, *z*_*hkl*_ corresponds to the cutting level along Cartesian *z* normal to the (*hkl*) glide plane.
